# Multi-Harmonic Source Localization Based on Sparse Component Analysis and Minimum Conditional Entropy

**DOI:** 10.3390/e22010065

**Published:** 2020-01-03

**Authors:** Yongzhen Du, Honggeng Yang, Xiaoyang Ma

**Affiliations:** College of Electrical Engineering, Sichuan University, Chengdu 610065, China; 2017223035119@stu.scu.edu.cn (Y.D.); pqlab99@126.com (H.Y.)

**Keywords:** sparse component analysis, conditional entropy, network impedance, independent component analysis, harmonic source localization

## Abstract

Aiming at the fact that the independent component analysis algorithm requires more measurement points and cannot solve the problem of harmonic source location under underdetermined conditions, a new method based on sparse component analysis and minimum conditional entropy for identifying multiple harmonic source locations in a distribution system is proposed. Under the condition that the network impedance is unknown and the number of harmonic sources is undetermined, the measurement node configuration algorithm selects the node position to make the separated harmonic current more accurate. Then, using the harmonic voltage data of the selected node as the input, the sparse component analysis is used to solve the harmonic current waveform under underdetermination. Finally, the conditional entropy between the harmonic current and the system node is calculated, and the node corresponding to the minimum condition entropy is the location of the harmonic source. In order to verify the effectiveness and accuracy of the proposed method, the simulation was performed in an IEEE 14-node system. Moreover, compared with the results of independent component analysis algorithms. Simulation results verify the correctness and effectiveness of the proposed algorithm.

## 1. Introduction

With the continuous penetration of new energy, power electronic equipment and non-linear loads, harmonic pollution in power systems is becoming increasingly serious. Harmonic source localization is the premise of harmonic source management and has important significance in the field of power quality [1,2,3,4,5].

Harmonic State Estimation (HSE) involves harmonic source localization. HSE is the focus of current research [4,5,6,7,8], including the least-squares method [4], singular value decomposition method [5], neural network [6], particle swarm algorithm [7], and Bayesian approach [8]. However, the above methods need to know the network topology and impedance parameters, which are actually changing in time or difficult to obtain. In addition, the above method needs to constantly adjust the position and quantity of the measurement node to meet the observability requirements of the approach, which is complicated. These factors limit the application in harmonic source localization.

In recent years, the Blind Source Separation (BSS) theory has provided new ideas for HSE. The Independent Component Analysis (ICA) method can locate the harmonic source under the condition that the network topology and harmonic impedance are unknown, avoiding the need for complete electrical parameters in traditional HSE methods [8,9,10]. In Reference [9,10], the ICA algorithm is used to separate the harmonic current waveform, and then the correlation between the harmonic current waveform and the voltage of system nodes is obtained. According to the value of the correlation, the harmonic source location is identified. However, ICA cannot resolve blind source separation in underdetermined situations. It requires a known number of harmonic sources to ensure that the number of Phase Measurement Unit (PMU) is greater than the source signals. However, in practical applications, the number of harmonic sources is often unknown. Therefore, the ICA algorithm has a significant limitation. Moreover, the Reference [9,10] does not consider the selection of the node harmonic voltage data. The input data of the ICA algorithm is random, which will also affect the accuracy of the result.

As an emerging blind source separation approach, Sparse Component Analysis (SCA) solves the problem of blind source separation in underdetermined situations and has been widely used in various fields in recent years [11,12,13,14]. Aiming at the problem that ICA requires many measurement nodes and cannot solve the problem of underdetermined blind source separation, a harmonic source localization method based on sparse component analysis is proposed. The measurement node configuration algorithm is used to select the location of the node that makes the harmonic separation current more accurate. The harmonic voltage data of these nodes is used as the input of SCA to accurately separate the harmonic current. Then the conditional entropy between the harmonic current and the system node is calculated, and the node corresponding to the minimum condition entropy is the location of the harmonic source. Simulation results verify the correctness and effectiveness of the proposed method.

## 2. Separation of Harmonic Currents in HSE Model

### 2.1. Relationship between HSE Model and BSS Model

The model of HSE is based on the harmonic voltage of the system bus. The harmonic source current of the injected node is the state variable which can be obtained through the harmonic impedance matrix. When the measurement error is ignored, the formula is as follows:

(1) $$U_{h}\left(t\right)=Z_{h}I_{h}\left(t\right)$$

where *t* is time and *h* is the order of harmonics. ***U****_h_*(*t*) is the harmonic voltage at time *t*, ***Z_h_*** is the impedance matrix of *M × N*-dimensions, and ***I****_h_*(*t*) is the harmonic current at time *t*.

The blind source separation model consists of the observed signal ***X***, the source signal ***S,*** and the mixing matrix ***A***. the model of BSS without noise can be described as:

(2) $$X(t)=A_{M\times N}S(t)t=1,2,\ldots ,T$$

where *M* and *N* are the numbers of observation signals and source signals, respectively. *T* is the number of sample points, ***S*** is the source signal matrix and ***X*** is the observation signal matrix.

By comparing Equations (1) and (2), it can be seen that there is a corresponding relationship between the HSE model and the BSS model: ***U****_h_*(*t*) corresponds to ***X***(*t*), ***Z****_h_* corresponds to ***A***, and ***I****_h_*(*t*) corresponds to ***S***(*t*). Therefore, the BSS model can be used in HSE applications. 

In Equation (2), for *M* ≥ *N*, the existing literature mainly uses a method based on the ICA. When *M* > *N*, it is called incomplete ICA, and *M* = *N* is the standard ICA. The Principal Component Analysis (PCA) transforms closely related variables into as few new variables as possible, making these new variables uncorrelated. That is, fewer comprehensive indicators are used to represent the information existing in each variable to achieve the effect of data dimensionality reduction [15,16]. For incomplete ICA, PCA is generally used to reduce the dimensionality of the observation signal to identify the number of independent components [17].

Since ICA can only solve the BSS problem in the case of *M* ≥ *N*, which requires the number of PMUs needs to be not less than the harmonic sources. For the underdetermined HSE problem, that is, the case of *M* < *N*, the application of ICA is limited. The existing research methods mainly use SCA [11,18,19,20].

After obtaining the HSE model, the solution of harmonic current ***S*** in ICA and SCA algorithms are discussed separately.

### 2.2. Independent Component Analysis Algorithm Using Fast-ICA

ICA algorithms mainly include Fast Independent Component Analysis (Fast-ICA) [21], information maximization method [22], and mutual information minimization method [23]. Among them, Fast-ICA, a fast fixed-point calculation based on negative entropy, is a commonly used algorithm for ICA [24,25], which has achieved good separation results.

First, preprocess the observation signals, that is, decentralise and whiten the observation signal, the objective function is constructed with an approximate expression of negative entropy. As follows:

(3) $$J(w)=[E\{G(w^{T}x\})-E\{G(v)\}{]}^{2}$$

where *w* is the separation vector in the demixing matrix ***W***, ${\left\|w\right\|}_{2}=1$; *x* is the pre-processed observation signal vector, and $w^{T}x$ is the separated source signal vector; *G* is a non-linear function whose first derivative is *g*; $G\left(x\right)=-1/a\exp\left(-ax^{2}/2\right)$, a≈1. *v* is a Gaussian random variable with a mean of 0 and a variance of 1.

The fast fixed-point ICA algorithm based on negative entropy is solved as follows:

(1) De-averaging and whitening of the observation signal ***X***;
(2) Identify the number of source signals n;
(3) Initialize the demixing matrix ***W***, $W={\left(w_{1},w_{2},\ldots ,w_{M}\right)}^{T}$;
(4) Iteratively calculate the demixing matrix ***W***: $W^{*}=E\{xg(W^{T}x)\}-E\{g^{\prime }W^{T}x\}W;$
(5) The orthogonalisation of the demixing matrix ***W***: $W={\left(W^{*}W^{*T}\right)}^{-1/2}W^{*}$;
(6) If ***W*** does not converge, return to step (4) and iterate until the convergence condition is satisfied.

After the ICA algorithm solves the optimal demixing matrix ***W***, the source signal is separated by direct calculation, that is:

(4) $$\hat{S}=WX$$

where the demixing matrix ***W*** is the inverse matrix of the mixing matrix ***A***.

It can be known from above that the blind source separation algorithm based on ICA needs to identify the number of source signals. In the actual application of harmonic state estimation, it is generally impossible to identify the number of harmonic sources in advance. In addition, ICA algorithm requires the number of measurement signals is not less than the number of source signals, that is, *M* ≥ *N*. In other words, it requires that the number of measurement devices is not less than the number of harmonic sources. When the measurement device is smaller than the number of harmonic sources, that is, *M* < *N*, Fast-ICA cannot achieve the separation of all harmonic source signals, so the ICA algorithm has a significant disadvantage.

For the non-singular mixing matrix ***A*** in ICA, the estimated value of the source signal $\hat{S}$ can be separated by obtaining the demixing matrix ***W***. However, for SCA when *M* < *N*, the inverse matrix of ***A*** does not exist, and the source signal cannot be separated by Equation (4). Therefore, a new method is needed to solve the mixed matrix ***A*** and the source signal ***S***, respectively.

### 2.3. Two-Step Method to Obtain the Mixing Matrix and Source Signal Respectively

In the case where the source signal is sparse in the time domain, clustering is used to separate the normalised mixing matrix ***A***. Suppose there are three sparse source signals *s*_1_, *s*_2_, *s*_3_, and two observation signals *x*_1_, *x*_2_, The mixed-signal *x* is obtained by matrix ***A***, as shown in Equation (5):

(5) $$\left[\begin{array}{c} x_{1}\\ x_{2} \end{array}\right]=\left[\begin{array}{ccc} a_{1,1} & a_{1,2} & a_{1,3}\\ a_{2,1} & a_{2,2} & a_{2,3} \end{array}\right]\left[\begin{array}{c} s_{1}\\ s_{2}\\ s_{3} \end{array}\right]$$

Since *s*_1_, *s*_2_, and *s*_3_ are sparse, at a certain time *t*, only the source signal *s_i_* appears in the mixed signal, and Equation (5) is simplified as:

(6) $$x_{1}\left(t\right)=a_{1,i}s_{i}\left(t\right),x_{2}\left(t\right)=a_{2,i}s_{i}\left(t\right)$$

Then, the scatter plot of the observed signal *x* will show the clustering direction *a_j_* of the harmonic source, which is a certain column vector of the mixing matrix. If a large number of observed signal points are clustered in the direction of the column vector of the mixing matrix ***A***, the mixing matrix ***A*** can be obtained by the clustering algorithm.

**Figure 1:**
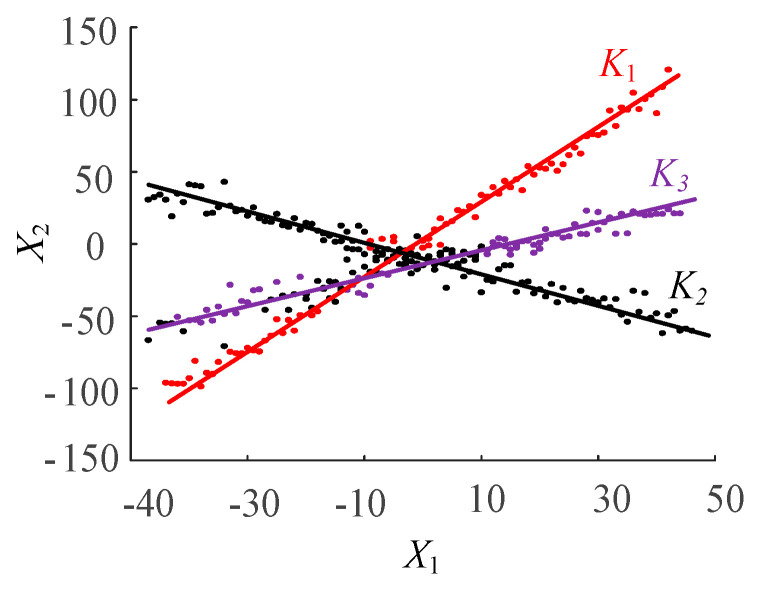
Clustering characteristics of measurement signals.

As shown in Figure 1, a scatter plot of a specific mixed signal is given. It can be seen that the clustering centre is very obvious for source signals that meet certain sparsity requirements. According to the slope of the three diagonal lines, combined with the normalization condition ${\left|\begin{array}{c} a_{i} \end{array}\right|}^{2}=1_{i}\left(i=1,2,3\right)$ of each column of the ***A*** matrix, the elements of the matrix ***A*** can be separated.

The SCA algorithm requires the source signal to be sparse, and the sparsity depends on the complexity of the signal rather than the number of sources [11]. For a few signals that do not have sparse signals in time domains, they need to be sparsely processed first [12,13,14]. In the existing literature, blind thinning is mainly used, and the sparse dictionary ***D*** is generated by wavelet transform [12,13] or short-time Fourier transform [11,14], and the source signal is transformed from the time domain to the wavelet domain or the frequency domain. The signal ***S*** is sparse, as shown in Equation (7):

(7) $$S^{T}=D{\left(S^{D}\right)}^{T}\;\Rightarrow \;S^{D}=S{\left(D^{-1}\right)}^{T}$$

where ***S*** is a signal that does not satisfy sparseness in the time domain, T represents the transpose of the matrix. ***S*^D^** is a sparseized signal; ***D*** is a complete sparse dictionary.

The sparse dictionary ***D*** is applied to the BSS model of Equation (2), it leads to the following equation:

(8) $$X{\left(D^{-1}\right)}^{T}=AS{\left(D^{-1}\right)}^{T}$$

From Equation (8), the sparse transform does not change the mixing matrix ***A***. After the thinning process, the source signal became ***S***(***D***^−1^)^T^ and the observed signal became ***X***(***D***^−1^)^T^. Then the sparse signal is linearly clustered, and the mixing matrix ***A*** can be obtained by calculating the slope of the line.

After obtaining the mixing matrix ***A***, the second step is to solve the underdetermined equation problem. In order to separate the harmonic current ***S***, the maximum posterior probability method is used. For source signals with sparsely distributed features, it can be assumed that the signal has the following Laplace probability density distribution function:

(9) $$p(s_{i})=\frac{1}{2\alpha }\exp(-\frac{\left|s_{i}\right|}{\alpha })$$

where *α* is the variance parameter, considering the uncertainty of the amplitude of the separated signal, it can be assumed that the source has the same variance parameter. Since the signals are independent of each other, the joint probability density of the source ***S*** is:

(10) $$\begin{array}{l} p(s)=\prod_{i=1}^{N}\frac{1}{2\alpha }\exp(-\frac{\left|s_{i}\right|}{\alpha })\\ \;=\frac{1}{2^{N}\alpha^{N}}\exp(-\frac{1}{\alpha }\sum_{i=1}^{N}\left|s_{i}\right|) \end{array}$$

Since ***A*** is known, maximizing the above equation under the conditions x=As, it can be given as:

(11) $$\begin{array}{l} \hat{s}=\arg\max(\frac{1}{2^{N}\alpha^{N}}\exp(-\frac{1}{\alpha }\sum_{i=1}^{N}\left|s_{i}\right|)\;|x=As)\\ \;=\arg\min[||s|{|}_{1}\;|x=As] \end{array}$$

The harmonic current ***S*** can be calculated by minimizing the above problem into a linear programming problem:

(12) $$\min\sum_{t=1}^{T}\sum_{j=1}^{N}\left|s_{j}(t)\right|\;\mathrm{given}\;\;x\left(t\right)=As\left(t\right)$$

According to Equation (12), under the limitation of x(t)=As(t), the separation process of the harmonic current is to minimize 1 norm for all sample points *x*(t), which is equivalent to the shortest path method [11].

## 3. Selection of Measurement Point Data

Considering the difference of node impedance in the topology of the power system, the shunting ability of different nodes is different, and the selection of measuring points will affect the accuracy. In order to make the harmonic waveform separated by the SCA algorithm as accurate as possible, it is necessary to select the harmonic voltage data of the node containing as much system topology and impedance information as possible. This paper uses a measurement node configuration method to select the measurement node.

From the perspective of graph theory, the power system network can be regarded as a graph *G*, g={V,E}, consisting of *n* nodes and *b* branches, where *V* represents a set of *n* nodes and E represents a set of b branches. The measurement network constitutes the subgraph $G^{\prime }=\left\{V^{\prime },E^{\prime }\right\}$ of *G* and has $V^{\prime }\subseteq V$, $E^{\prime }\subseteq E$. If graph *G* and graph $G^{\prime }$ satisfy $V\subseteq V^{\prime }$ at the same time, the power network system topology is observable.

The numerical analysis method mainly judges the observability by the triangulation of the impedance matrix to find out whether there is a zero principal element, the calculation amount is large and the precision is susceptible to the cumulative error. In this paper, the sparseness of the node admittance matrix is used to select the measurement nodes.

**Figure 2:**
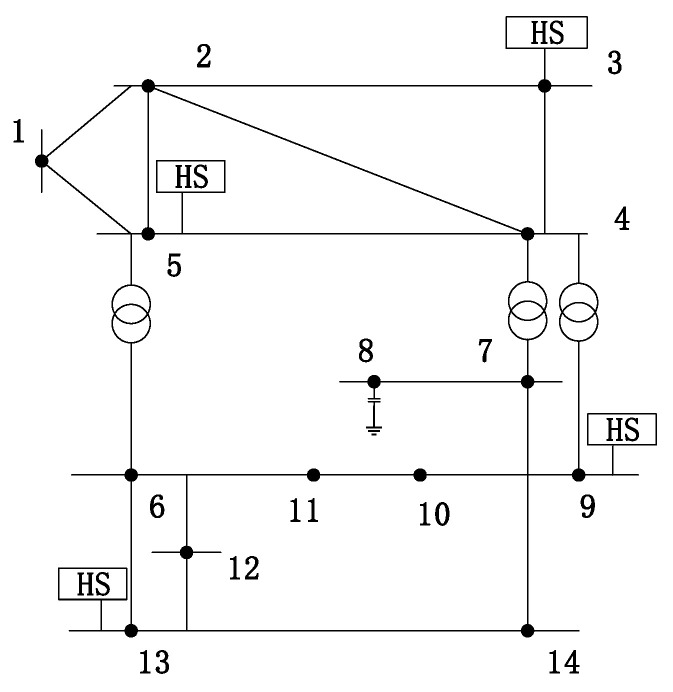
IEEE 14-node system topology.

As shown in Figure 2, the IEEE 14-node system is taken as an example. Among them, the nodes 1–14 constitute a set ***V***, and the connection line between the nodes constitutes ***E***. The main steps of configuration are as follows:

(1) Firstly, construct the system association matrix ***R***, and define the elements in ***R*** as follows:
  (13) $$R_{i,j}=\{\begin{array}{c} 1\\ 1\\ 0 \end{array}\begin{array}{c} \mathrm{Node}\;i\;\mathrm{and}\;\mathrm{node}\;j\;\mathrm{are}\;\mathrm{the}\;\mathrm{same}\;\mathrm{node}\\ \mathrm{Node}\;i\;\mathrm{is}\;\mathrm{connected}\;\mathrm{to}\;\mathrm{node}\;j\\ others \end{array}$$
  According to the definition of ***R***, the association matrix of IEEE 14-nodes can be obtained as follows:
  (14) $$R_{14,14}=\left[\begin{array}{ccccc} 1 & 1 & \ldots & 0 & 0\\ 1 & 1 & \cdots & 0 & 0\\ \vdots & \vdots & \cdots & \vdots & \vdots \\ 0 & 0 & \cdots & 1 & 1\\ 0 & 0 & \cdots & 1 & 1 \end{array}\right]$$
(2) In order to ensure the optimal configuration of the nodes, it is necessary to have at least one measuring device for each row in Equation (14). According to the association matrix of Equation (14), the following relationship is obtained:
  (15) $$\{\begin{array}{c} f_{1}=x_{1,1}+x_{1,2}+x_{1,5}\geq 1\\ f_{2}=x_{2,1}+x_{2,2}+x_{2,3}+x_{2,4}+x_{2,5}\geq 1\\ \vdots \\ f_{13}=x_{13,6}+x_{13,12}+x_{13,13}+x_{13,14}\geq 1\\ f_{14}=x_{14,9}+x_{14,13}+x_{14,14}\geq 1 \end{array}$$
  where “+” is the logical operation “OR”, $f_{i}\geq 1$ indicating that there is at least one non-zero value in the *i*-th row of the association matrix ***R***.

The objective function expression is:

(16) $$\min N_{tatal}=\sum_{i}^{n}\delta_{i}$$

where $\delta_{i}$ is the condition of whether the measuring device is installed in the *i*-th node. $\delta_{i}=1$ indicating that the node has a PMU device, $\delta_{i}=0$ indicating that the node has no PMU device. *n* is the number of nodes of the system and $N_{total}$ is the total number of PMU of the system.

## 4. Identify the Location of the Harmonic Source Using Minimum Conditional Entropy

The conditional entropy of two random variables is a measure of the interdependency. Comparing with the correlation coefficient, it is a better way to measure the dependence between two random variables [26].

The discrete form of the entropy equation is:

(17) $$H(X)=-\sum_{x=1}^{N}p(x)\log_{2}p(x)$$

where *X* is a random variable, *x* is an event, and *p*(*x*) is a probability density function.

To determine the uncertainty of variable *A* after the occurrence of the variable *B*, conditional entropy is used. Assume that the random variables *A* and *B* are composed of *N a_i_* and *b_i_* elements, respectively. For *i* = 1, 2,…, *N*, the conditional entropy can be defined as:

(18) $$H(A|b_{i})=-\sum_{i=1}^{N}p(a_{i}|b_{i})\log_{2}p(a_{i}|b_{i})$$

The conditional entropy of the variables *A* and *Β* can be written as:

(19) $$H(A|B)=\sum_{i=1}^{N}p(b_{i})H(A|b_{i})$$

The stronger the interdependence between two random variables, the smaller their conditional entropy. Due to the shunting effect of the power system, the correlation between the harmonic current and the harmonic voltage of the injection node is higher than that of the non-injected node [9,10], which has the minimum conditional entropy. Therefore, by comparing the conditional entropy of all nodes in the system, the location of the harmonic source can be identified.

## 5. Harmonic Source Localization Using CA and Minimum Conditional Entropy

The multi-harmonic source localization process based on SCA and minimum conditional entropy is as follows:

(1) Measuring system node harmonic voltage
  Measure the harmonic voltage *U_h_* of all nodes in the system.
(2) Selection of measurement points
  According to the network topology of the power system, a measurement node configuration model is established to determine the location and quantity of the measurement nodes.
(3) Sparsification of harmonic voltage signals
  Using the proposed thinning method, thinning the harmonic voltage signal of the measurement node.
(4) Separation of harmonic current
  The harmonic voltage of these measurement nodes is used as the input of the SCA separation algorithm. The hybrid matrix ***A*** and the harmonic current ***S*** is obtained.
(5) Localization of the harmonic sources

Starting from the first separated harmonic current, the conditional entropy between the harmonic current and the harmonic voltage of all nodes is calculated, and the node corresponding to the minimum conditional entropy is found, that is, the location of the harmonic source. Identify the node where each harmonic current is located and complete the location of the harmonic source.

**Figure 3:**
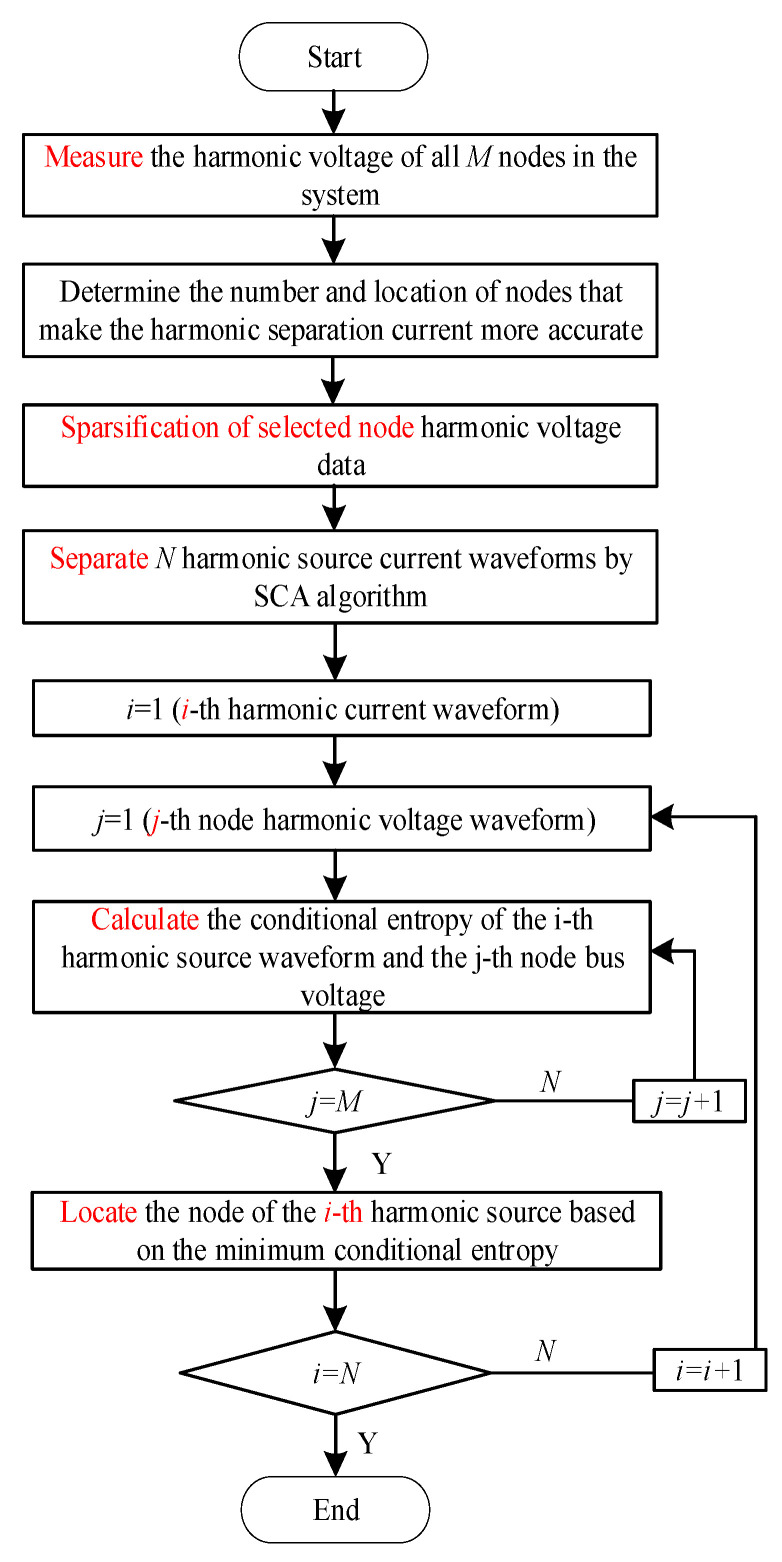
Process of the harmonic source localization algorithm.

In summary, the process of the proposed algorithm for harmonic source localization can be illustrated in Figure 3.

## 6. Example Test

In order to verify the effectiveness of the proposed method, an IEEE 14-node system was selected for simulation. As shown in Figure 2, there are 4 Harmonic Sources (HS) in the simulation, which are located at nodes 3, 5, 9, and 13 respectively. The harmonic current adopts the 5th and 7th harmonic typical curves [27], which have a total of 1000 sampling points and are superimposed with a random disturbance of ±5% on the source signal. Under the condition that the system network topology and system impedance are unknown, the proposed algorithm is analyzed.

### 6.1. Comparison between SCA and Fast-ICA Configuration Schemes

In order to analyze the effectiveness of the SCA algorithm, it is compared with the Fast-ICA algorithm. The Fast-ICA algorithm cannot solve the problem of BSS in the underdetermined situation, therefore, in order to avoid the omission of the harmonic sources, the number of harmonic sources needs to be identified. The simulation system has 4 harmonic sources in total, so Fast-ICA requires at least 4 PMUs, and the SCA algorithm theoretically does not delimit the number of measurement devices. In order to ensure the accuracy of the SCA algorithm, the number of measuring devices is at least two. *N*_i_ indicates that the PMU is located at node *i*. The SCA algorithm selects nodes based on the optimal measurement node configuration results. And randomly selects the harmonic voltages of 4 nodes as the measurement nodes of ICA. The comparison between SCA and Fast-ICA configuration schemes is as follows.

As can be seen from Table 1, compared with Fast-ICA, SCA does not need to determine the number of source signals in advance and can solve the problem of blind source separation in underdetermined situations, which avoids determining the number of source signals before the separation of harmonic signals. In addition, the ICA algorithm requires that the number of measurement devices is not less than the number of harmonic sources. To meet this condition, it is required a large number of measurement devices. Otherwise, all harmonic current signals cannot be separated. Considering the economics of the measurement device configuration, the SCA algorithm is more advantage, which is easier to apply on a large scale in practice.

### 6.2. Accuracy Analysis of Separating Harmonic Current Waveform and Actual Harmonic Current

The harmonic voltage data of nodes 2, 6, and 9 are taken as the input of the SCA algorithm. The harmonic current is separated and normalized by the SCA algorithm. The separated waveforms are sorted by the correlation coefficient to determine the harmonics corresponding to the harmonic current injection node. Comparing with the normalized actual harmonic source current waveform, the results are as follows.

**Figure 4:**
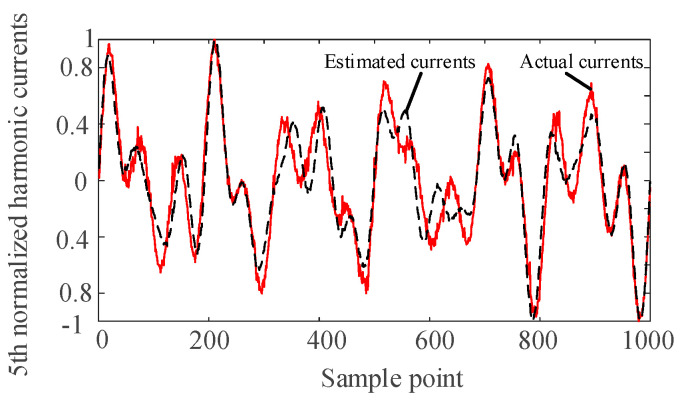
Normalized estimated harmonic current and actual harmonic current at injection node 1.

**Figure 5:**
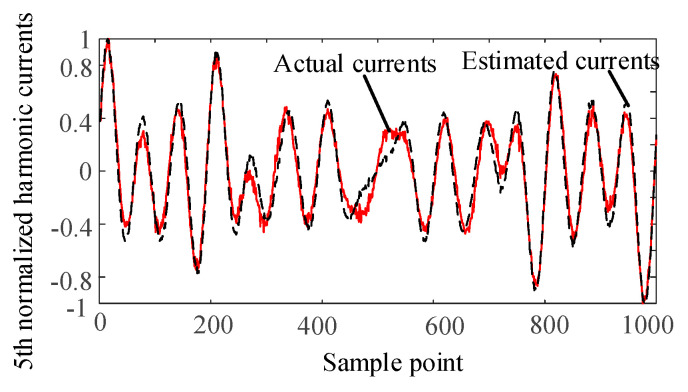
Normalized estimated harmonic current and actual harmonic current at injection node 2.

**Figure 6:**
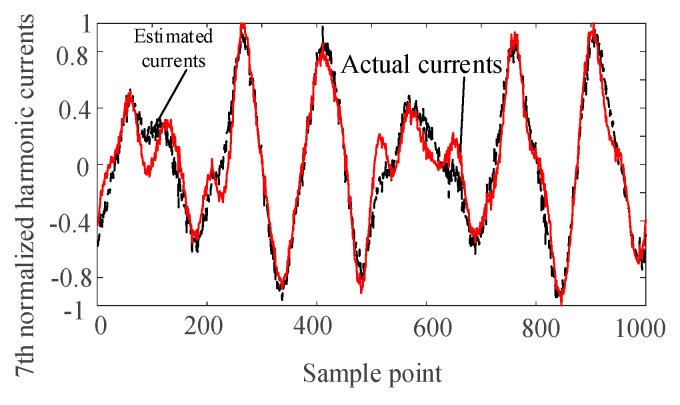
Normalized estimated harmonic current and actual harmonic current at injection node 3.

**Figure 7:**
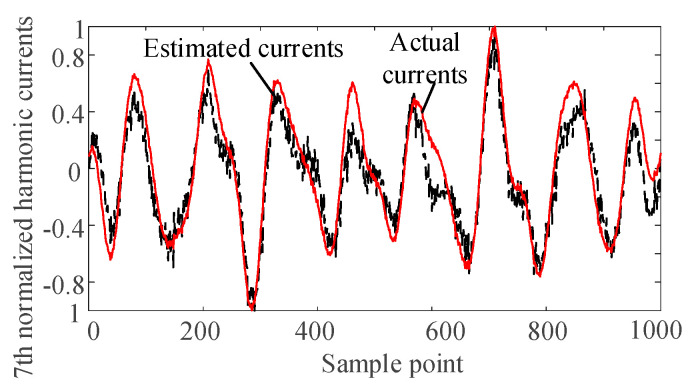
Normalized estimated harmonic current and actual harmonic current at injection node 4.

It can be seen from Figure 4, Figure 5, Figure 6 and Figure 7 that the separated harmonic current curve is highly consistent with the actual harmonic curve. The separated harmonic current waveforms accurately restore the actual current, which indicates that the SCA algorithm is accurate and effective.

### 6.3. Performance Comparison between SCA and Fast-ICA Algorithms 

In order to better reflect the error between the separated value and the actual value, the correlation coefficient between the separated value and the actual value, the Mean Absolute Error (MAE) and the Root Mean Square Error (RMSE) are selected to quantify the separation error:

(20) $$MAE=\frac{1}{T}\sum_{i=1}^{T}\left|y_{i}-x_{i}\right|$$

(21) $$RMSE=\sqrt{\frac{1}{T}\sum_{i=1}^{T}{\left(x_{{}^{i}}-y_{{}^{i}}\right)}^{2}}$$

where *y_i_* and *x_i_* represent the separation value and the actual value of the harmonic current at the time *t* respectively. And *T* is the total number of samples. The MAE characterizes the mean of the absolute error of the separated and actual values, and the RMSE is used to quantify the deviation between the separated and actual values.

In order to analyze the accuracy of the SCA algorithm, it is compared with the Fast-ICA algorithm. The correlation coefficients, MAE and RMSE of the harmonic current and actual current separated by SCA and Fast-ICA are calculated separately. The error of the two are shown in Table 2, Table 3 and Table 4:

From Table 2, Table 3 and Table 4, it can be seen that the harmonic current of SCA is closer to 1, and the MAE and RMSE are smaller than that of Fast-ICA. It shows that compared with Fast-ICA, which randomly selects measurement nodes, the SCA algorithm has higher accuracy of the separated harmonic current waveforms, and the SCA algorithm does not need to determine the number of harmonic sources first. In conclusion, the SCA algorithm in the paper requires fewer prerequisites but has better accuracy.

### 6.4. Identifying the Location of Harmonic Sources Using Minimum Conditional Entropy

**Figure 8:**
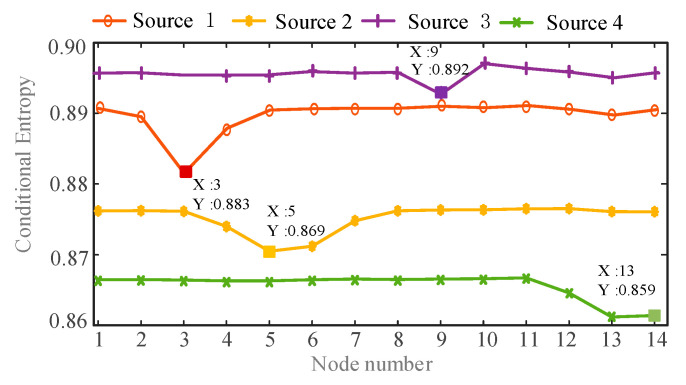
The conditional entropy between estimated harmonic currents and measured harmonic voltages at *h* = 5.

**Figure 9:**
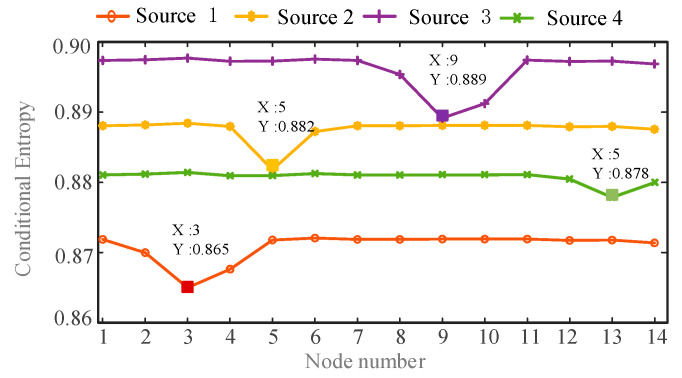
The conditional entropy between estimated harmonic currents and measured harmonic voltages at *h* = 7.

In order to identify the location of the harmonic source, the conditional entropy between each harmonic current and harmonic voltage of all nodes is calculated. Then the minimum value of conditional entropy is found. In order to facilitate the analysis results, draw a line graph of the conditional entropy between harmonic currents and the harmonic voltages of all nodes, as shown in Figure 8 and Figure 9:

From the comparison of Figure 8 and Figure 9, it can be seen that the harmonic source 1 has the minimum conditional entropy at the node 3, the harmonic source 2 has the minimum conditional entropy at the node 5, and the harmonic source 3 has the minimum conditional entropy at the node 9. The harmonic source 4 has the minimum conditional entropy at the node 13. Therefore the harmonic sources are located at nodes 3, 5, 9, and 13 respectively, which are consistent with the actual harmonic source locations. The results verify the validity and accuracy of the proposed method.

## 7. Conclusions

(1) A multi-harmonic source localization method based on sparse component analysis and minimal conditional entropy is proposed in this paper, which solves the problems of harmonic separation in underdetermined situations. The measurement node configuration algorithm is applied to select the input data of SCA; then, the source signal is sparsely obtained to calculate the mixed impedance ***A*** and the harmonic current ***S***. Compared with Fast-ICA, there is no need to determine the number of harmonic sources in advance, and fewer measurement devices are required.

(2) The proposed method can only measure the harmonic voltage of the node when the network topology and harmonic impedance are unknown. Simulation results show that the location of the harmonic source is accurately located, which verify the validity and accuracy of the proposed method.

(3) This paper only considers the effects of weak additive noise. Under severe noise conditions, the performance of the existing SCA algorithm decreases significantly. How to accurately isolate the harmonic current waveform in the case of harsh noise is the next step of research.

## Figures and Tables

**Table 1:** Configuration results of the Sparse Component Analysis (SCA) and Fast Independent Component Analysis (Fast-ICA).

Algorithm	SCA	Fast-ICA
Underdetermined blind source separation	Yes	No
Determine the number of source signals in advance	No	Yes
Number of measurement points required	≥2	≥4
Measurement configuration cost	Low	High
Configuration scheme	*N*_2_, *N*_6_, *N*_9_	*N*_2_, *N*_6_, *N*_9_, *N*_12_

**Table 2:** Coefficient between actual and estimated currents.

Injection Node	Harmonic Number	Correlation Coefficient	
		SCA	Fast-ICA
Injection node 1	5	0.9374	0.9300
	7	0.9762	0.9721
Injection node 2	5	0.9723	0.9655
	7	0.9601	0.9593
Injection node 3	5	0.9733	0.9834
	7	0.9682	0.9651
Injection node 4	5	0.9707	0.9677
	7	0.9447	0.9226

**Table 3:** Mean Absolute Error (MAE) between actual and estimated currents.

Injection Point	Harmonic Number	MAE	
		SCA	Fast-ICA
Injection node 1	5	0.1248	0.1358
	7	0.1096	0.1226
Injection node 2	5	0.0801	0.1521
	7	0.1031	0.1323
Injection node 3	5	0.0928	0.1316
	7	0.0912	0.1912
Injection node 4	5	0.0883	0.1833
	7	0.1305	0.1402

**Table 4:** Root Mean Square Error (RMSE) between actual and estimated currents.

Injection Point	Harmonic Number	RMSE	
		SCA	Fast-ICA
Injection node 1	5	0.1482	0.1538
	7	0.1143	0.1234
Injection node 2	5	0.0941	0.1231
	7	0.1081	0.1223
Injection node 3	5	0.1452	0.1466
	7	0.1275	0.1273
Injection node 4	5	0.1098	0.1123
	7	0.1553	0.1562

## References

[1] Saxena D., Bhaumik S., Singh S.N. (2014). Identification of Multiple Harmonic Sources in Power System Using Optimally Placed Voltage Measurement Devices. IEEE Trans. Ind. Electron..

[2] Melo I.D., Pereira J.L., Ribeiro P.F., Variz A.M., Oliveira B.C. (2019). Harmonic state estimation for distribution systems based on optimization models considering daily load profiles. Electr. Power Syst. Res..

[3] Ardakanian O. (2019). Leveraging Sparsity in Distribution Grids: System Identification and Harmonic State Estimation. ACM SIGMETRICS Perform. Eval. Rev..

[4] Meliopoulos A.P.S., Zhang F., Zelingher S. (1994). Power system harmonic source estimation. IEEE Trans. Power Deliv..

[5] Heydt G.T. (1989). Identification of harmonic sources by a state estimation technique. IEEE Trans. Power Deliv..

[6] Lin W.M., Lin C.H., Tu K.P., Wu C.H. (2005). Multiple harmonic source detection and equipment identification with cascade correlation network. IEEE Trans. Power Deliv..

[7] Lu Z., Ji T.Y., Tang W.H., Wu Q.H. (2008). Optimal Harmonic Estimation Using a Particle Swarm Optimizer. IEEE Trans. Power Deliv..

[8] D’Antona G., Muscas C., Sulis S. (2009). State estimation for the localization of harmonic sources in electric distribution systems. IEEE Trans. Instrum. Meas..

[9] Zang T., He Z., Fu L., Chen J., Qian Q. (2016). Harmonic Source Localization Approach Based on Fast Kernel Entropy Optimization ICA and Minimum Conditional Entropy. Entropy.

[10] Farhoodnea M., Mohamed A., Shareef H. A new method for determining multiple harmonic source locations in a power distribution system. Proceedings of the IEEE International Conference on Power & Energy.

[11] Bofill P., Zibulevsky M. (2001). Underdetermined blind source separation using sparse representations. Signal Process..

[12] Yu X., Xu J., Hu D., Xing H. (2013). A new blind image source separation algorithm based on feedback sparse component analysis. Signal Process..

[13] Li Y., Amari S.I., Cichocki A., Ho D.W., Xie S. (2006). Underdetermined blind source separation based on sparse representation. IEEE Trans. Signal Process..

[14] Reju V.G., Koh S.N., Soon I.Y. (2009). An algorithm for mixing matrix estimation in instantaneous blind source separation. Signal Process..

[15] Yunusa-Kaltungo A., Sinha J.K. (2016). Generic vibration-based faults identification approach for identical rotating machines installed on different foundations, VIRM 11-Vibrations in Rotating. Machinery.

[16] Yunusa-Kaltungo A., Sinha J.K. (2016). Sensitivity analysis of higher order coherent spectra in machine faults diagnosis. Struct. Health Monit..

[17] Jolliffe I.T. (2002). Principal Component Analysis.

[18] Georgiev P., Theis F., Cichocki A. (2005). Sparse component analysis and blind source separation of underdetermined mixtures. IEEE Trans. Neural Netw..

[19] Yang Y., Nagarajaiah S. (2013). Output-only modal identification with limited sensors using sparse component analysis. J. Sound Vib..

[20] Xu Y., Brownjohn J.M., Hester D. (2019). Enhanced sparse component analysis for operational modal identification of real-life bridge structures. Mech. Syst. Signal Process..

[21] Hyvarinen A. (1999). Fast and robust fixed-point algorithms for independent component analysis. IEEE Trans. Neural Netw..

[22] Bell A.J., Sejnowski T.J. (1995). An information-maximization approach to blind separation and blind deconvolution. Neural Comput..

[23] Amari S.I., Cichocki A., Yang H.H. (1996). A new learning algorithm for blind signal separation. Advances in Neural Information Processing Systems.

[24] Langlois D., Chartier S., Gosselin D. (2010). An introduction to independent component analysis: InfoMax and FastICA algorithms. Tutor. Quant. Methods Psychol..

[25] Behera S.K. (2009). Fast ICA for Blind Source Separation and Its Implementation. Ph.D. Dissertation.

[26] Li W. (1990). Mutual information functions versus correlation functions. J. Stat. Phys..

[27] Gursoy E. (2008). Harmonic load identification using complex independent component analysis. IEEE Trans. Power Deliv..

